# The Effects of High Level Magnesium Dialysis/Substitution Fluid on Magnesium Homeostasis under Regional Citrate Anticoagulation in Critically Ill

**DOI:** 10.1371/journal.pone.0158179

**Published:** 2016-07-08

**Authors:** Mychajlo Zakharchenko, Ferdinand Los, Helena Brodska, Martin Balik

**Affiliations:** 1 Dept. of Anesthesiology and Intensive Care, First Faculty of Medicine, Charles University and General University Hospital in Prague, Prague, Czech Republic; 2 Dept. of Clinical Biochemistry, First Faculty of Medicine, Charles University and General University Hospital in Prague, Prague, Czech Republic; Azienda Ospedaliero-Universitaria Careggi, ITALY

## Abstract

**Background:**

The requirements for magnesium (Mg) supplementation increase under regional citrate anticoagulation (RCA) because citrate acts by chelation of bivalent cations within the blood circuit. The level of magnesium in commercially available fluids for continuous renal replacement therapy (CRRT) may not be sufficient to prevent hypomagnesemia.

**Methods:**

Patients (n = 45) on CRRT (2,000 ml/h, blood flow (Qb) 100 ml/min) with RCA modality (4% trisodium citrate) using calcium free fluid with 0.75 mmol/l of Mg with additional magnesium substitution were observed after switch to the calcium-free fluid with magnesium concentration of 1.50 mmol/l (n = 42) and no extra magnesium replenishment. All patients had renal indications for CRRT, were treated with the same devices, filters and the same postfilter ionized calcium endpoint (<0.4 mmol/l) of prefilter citrate dosage. Under the high level Mg fluid the Qb, dosages of citrate and CRRT were consequently escalated in 9h steps to test various settings.

**Results:**

Median balance of Mg was -0.91 (-1.18 to -0.53) mmol/h with Mg 0.75 mmol/l and 0.2 (0.06–0.35) mmol/h when fluid with Mg 1.50 mmol/l was used. It was close to zero (0.02 (-0.12–0.18) mmol/h) with higher blood flow and dosage of citrate, increased again to 0.15 (-0.11–0.25) mmol/h with 3,000 ml/h of high magnesium containing fluid (p<0.001). The arterial levels of Mg were mildly increased after the change for high level magnesium containing fluid (p<0.01).

**Conclusions:**

Compared to ordinary dialysis fluid the mildly hypermagnesemic fluid provided even balances and adequate levels within ordinary configurations of CRRT with RCA and without a need for extra magnesium replenishment.

**Trial Registration:**

ClinicalTrials.gov Identifier: NCT01361581

## Introduction

The magnesium concentrations of current commercially available fluids designed for renal replacement therapy (CRRT) are equal to plasmatic normal range (0.7–1.0 mmol/l)[1]. This originates from expected fluid application in advanced renal failure with magnesium cummulation and its high levels. CRRT in critically ill is usually indicated in earlier phase of renal insufficiency based on complex indication criteria [2, 3]. At this time the levels of magnesium are mostly normal or only mildly elevated. The daily magnesium substitution is a matter of debate with regards to reported deficit of intracellular magnesium in general population and in intensive care patients in particular [4–8]. Thus CRRT in ICU frequently requires early magnesium substitution and the requirements are further increased with regional citrate anticoagulation (RCA)[7, 9].

**Fig 1:**
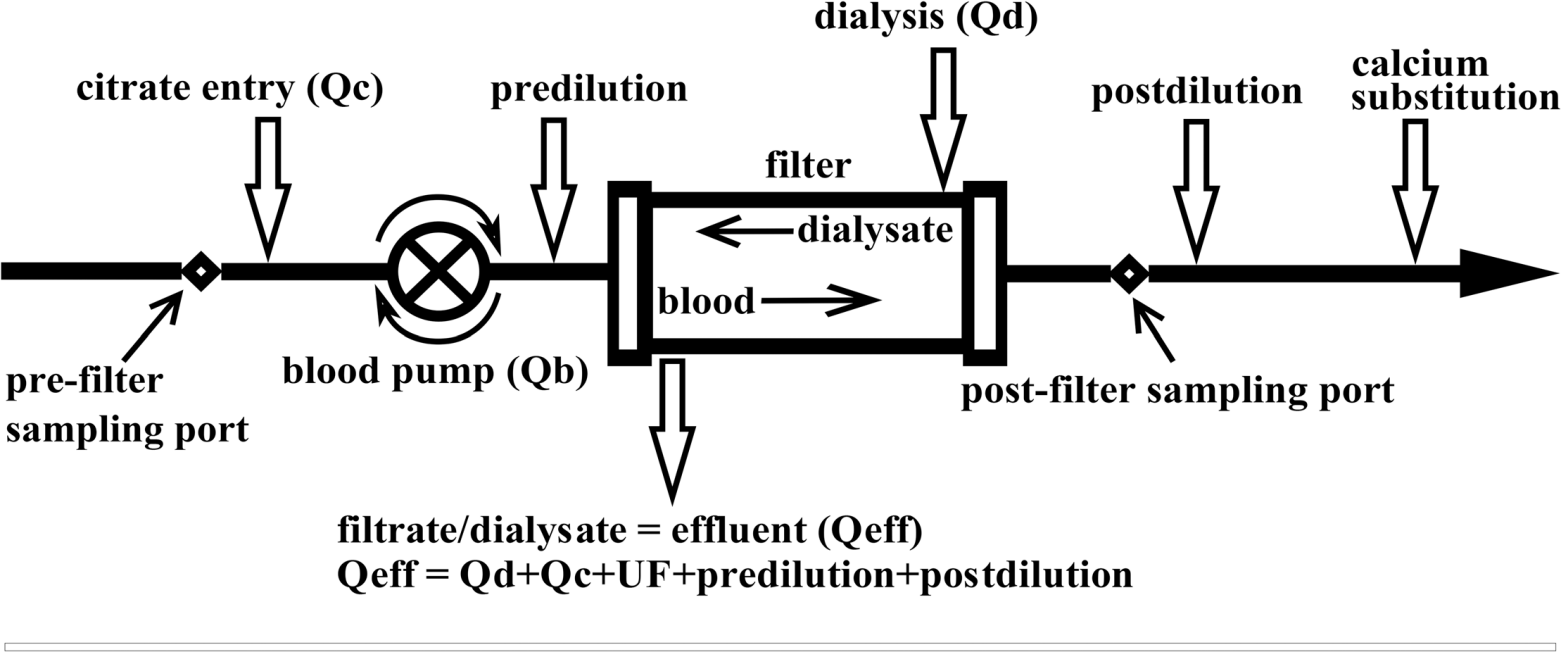
Configuration of the CRRT circuit under citrate anticoagulation. Qb, blood flow (L/h); Qc—4% citrate flow (L/h); Qd—dialysis flow (L/h); Qeff,—effluent flow (L/h); UF indicates ultrafiltration, that is, net fluid removal (L/h).

Compared to heparin, RCA showed almost no bleeding and superior circuit life enabling the delivery of high quality CRRT [10–13]. The cumulative evidence suggesting efficacy and safety of RCA has been reflected in the guidelines promoting the use of citrate in preference to standard heparin for prevention of filter clotting even in patients without an increased bleeding risk [14]. Citrate inhibits coagulation through chelation of ionised calcium (Ca^2+^) which is the principle of all citrate modes of regional anticoagulation. The postfilter decrease of ionized calcium is used to guide the prefilter dosage of citrate and substitution of calcium at venous end of blood tubing is required to maintain systemic level of Ca^2+^(Fig 1).

Various citrate protocols has shown either mild cummulation, deficit or even balance of total calcium mostly depending on the intensity of calcium substitution [15–17]. RCA is always accompanied by meticulous calcium substitution, much less attention is paid to changes and substitution of magnesium which is chelated by citrate similarly to calcium [7, 9]. The balance of total magnesium (Mg^tot^) is not well explored under various modalities and solutions used with citrate anticoagulation. Brain found a significantly negative magnesium balance using dialysis/replacement fluids containing 0.5 mmol Mg^tot^ per liter [7]. In our previous research on ionised magnesium we found similar negative magnesium balance when using fluids with 0.75 mmol/l Mg^tot^ per liter [9]. With regards to rather difficult assessment of magnesium deficit in critically ill RCA may lead to inapparent losses of magnesium and depletion of its relatively low body pool [6, 8, 18]. Deficit of Mg^tot^ is related to cardiovascular stability, pulmonary hypertension, resistance to insulin, neuromuscular function as well as to non-recovery of renal function and mortality of patients [19, 20].

In our research we developed and tested novel fluid for RCA containing high level magnesium [21]. Our hypothesis was that the amount of magnesium in commonly used dialysis/substitution fluids should be supranormal for application with citrate modality in the intensive care setting. We hypothesised that the novel fluid would be suitable to compensate for the losses during citrate modality without need for additional magnesium supplementation. Our aim was to test novel high level magnesium fluid within ordinary range of citrate dosage related to blood flow and within ordinary range of CRRT dosage. Eliminating a need for parenteral magnesium replenishment may contribute to patient safety and also to simplicity and cost effectivity of RCA modalities.

## Materials and Methods

The research was performed as a single center prospective 27h sequential exposure cohort study at 20 bed ICU of University Hospital [21]. The study received approval from the Ethics Committee of the General University Hospital, written informed consent was obtained from next of kin. All patients were included between December 2012 and February 2014.

The fluxes of magnesium and calcium and possible relationships to citrate dosage and citratemias were studied during postdilution continuous venovenous hemodiafiltration (CVVHDF) and continuous venovenous haemofiltration (CVVH) performed on Aquarius device (Baxter®, Irvine, CA, USA) with 1.9 m^2^ polysulfone filter (Aquamax®, Bellco, Mirandola, Italy) (Fig 1). Indications for renal replacement therapy were renal failure with elevated levels of uremic toxins and loss of response to diuretics. Prescribed starting CRRT dosage was 2000 ml/h (20–25 ml/kg/h) [22, 23], ultrafiltration (net fluid loss) was titrated according to the haemodynamic needs throughout the study.

All substitution in CVVHDF modality was given as postdilution and divided equally between predilution and postdilution in CVVH modality (see Fig 1). The blood flow (Qb) was set initially at 100 ml/min in both modalities. Reason to use a lower Qb was to allow for a lower citrate dose, which prevents metabolic alkalosis and hypernatremia [24, 25]. A Qb of 100 ml/min is sufficient to saturate dialysate at flow rate of 2 L/hour [26]. 4%TSC infusion was initiated at 200 ml/h and titrated in increments to maintain the postfilter Ca^2+^ under 0.4 mmol/l. Ca^2+^ was checked every hour until stable and thereafter 3-hourly. Calcium chloride (10%) was infused into a port distal from the venous bubble trap to maintain arterial Ca^2+^ within normal range (0.85–1.2 mmol/l). Arterial Ca^2+^ was monitored every 6 hours.

The magnesium balances when using newly designed original calcium-free high magnesium (1.50 mmol/l) containing solution for RCA [21] were compared to the RCA with ordinary calcium-free fluid containing 0.75 mmol/l of magnesium. As part of the registered research project on metabolic impact of regional citrate anticoagulation (NCT01361581) the authors had developed a novel lactate based fluid [21] and later commenced with its routine use. The novel fluid with high level of magnesium has become a part of standard patient care. Nevertheless, the effects of the novel fluid upon compensation of magnesium losses [9] and hypomagnesemia have not been published yet.

**Fig 2:**
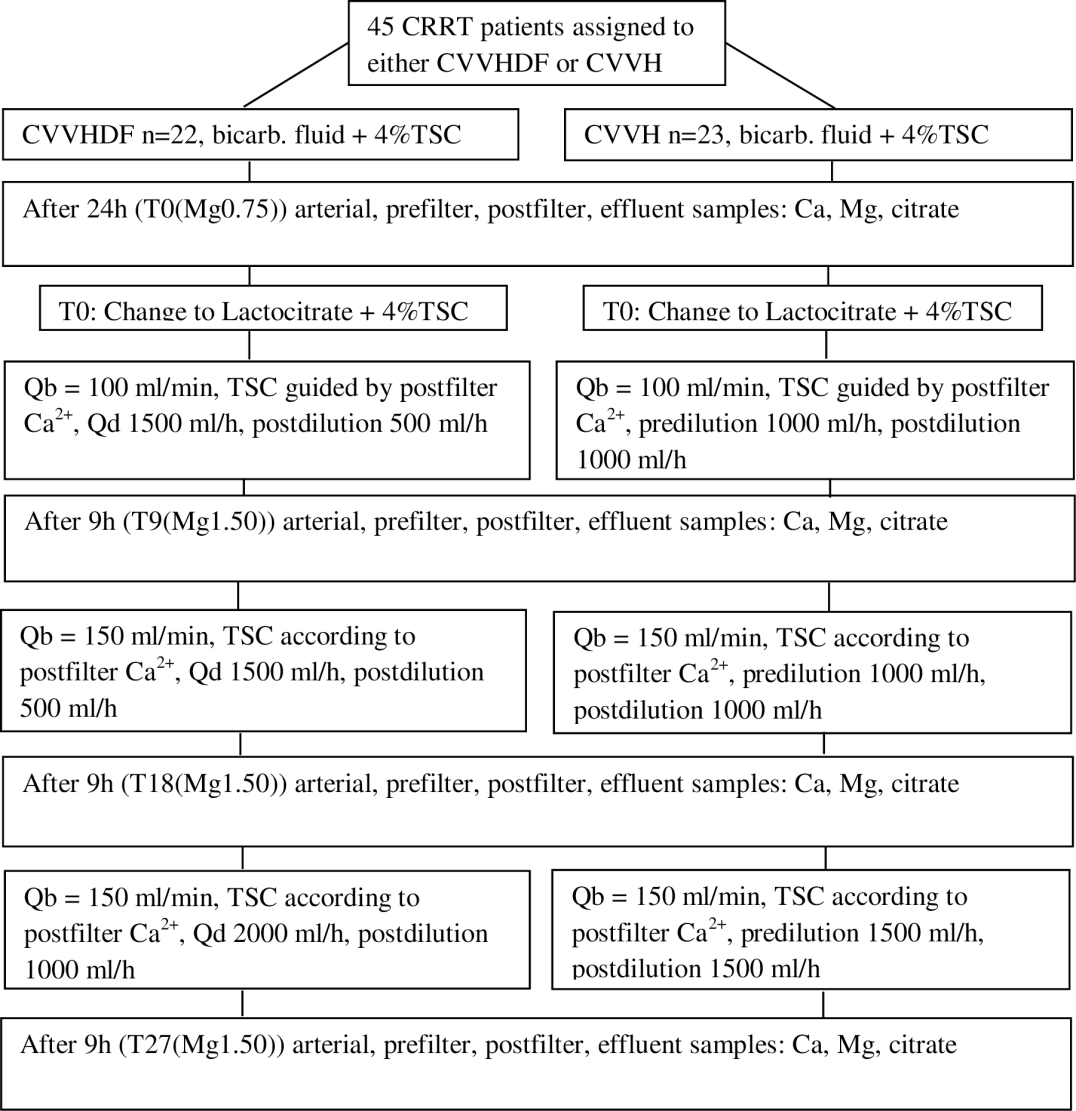
Flow chart of the 27 hour sequential exposure study. Ultrafiltration (i.e. net fluid removal) was always administered according to haemodynamic needs (4%TSC trisodium citrate, Qb blood flow, Qd dialysis flow).

The patients on CVVHDF or CVVH were started with standard magnesium concentration bicarbonate buffered solution with reduced levels of sodium and bicarbonate (Citralysate®, GML, Czech Republic, Na 133 mmol/l, K 2.0 mmol/l, Mg 0.75 mmol/l, Cl 116.5 mmol/l, glucose 5.6 mmol/l, HCO_3_^-^ 20 mmol/l) [25]. The 27h study commenced with first sampling at T0(Mg0.75) (see Fig 2) after at least 24h of CRRT. The patients on CVVHDF and CVVH were then switched to the fully certified (CE mark) novel lactate based fluid with reduced sodium and lactate („Lactocitrate“, Na 130, K 2.0, Cl 116, Mg 1.5, P 1.0, glucose 5.5, lactate 18 mmol/l) [21]. Both solutions were used as dialysis and replacement fluids in CVVHDF and CVVH (Fig 2). After the change for fluid with Mg 1.50 mmol/l at T9(Mg1.50) the Qb was increased to 150 ml/min with a corresponding increase of 4%TSC. After sampling at 18 hours (T18(Mg1.50)) the increased Qb with higher dosage of 4%TSC was maintained and dosage of the novel fluid was increased to 3000 ml/h to test the effects of a higher dose upon ion levels and balances. Last sampling was taken at 27 hours of the study (T27(Mg1.50)). The reasons for monitoring of the ion fluxes under increased blood flow (Qb) associated with higher dosage of citrate and later with escalated dosage of the novel hypermagnesemic fluid were to see the performance of the fluid during various ordinary settings of CRRT [21].

A routine substitution of intravenous 20–30 ml of 20% magnesium sulfate (16.2–24.3 mmol) per day was part of the protocol when Mg 0.75 mmol/l fluid was applied. Besides ordinary enteral and parenteral nutrition no extra magnesium was administered after switch to the Mg 1.50 mmol/l containing fluid.

Samples at T0(Mg0.75), T9(Mg1.50), T18(Mg1.50) and T27(Mg1.50) were drawn from the arterial blood, the effluent, the prefilter and postfilter ports of the circuit (see Figs 1 and 2). Besides total calcium and magnesium the laboratory analysis consisted of citrate levels (measured by capillary zone electrophoresis, P/ACE 5100, Beckman). The normal level of magnesium taken by photometry (Cobas-Integra analyser) in the hospital biochemical laboratory is 0.7–1.0 mmol/l.

### Calculation of magnesium and calcium fluxes from patient´s CRRT circuit to the effluent

The amount of magnesium removed across the filter was deducted from magnesium delivered as part of dialysis fluid and postdilution. Effluent removal of magnesium was calculated from effluent flow (Qeff) multiplied by magnesium concentration in effluent ([Mg]eff). Magnesium input was calculated as dialysis flow (Qd) times magnesium concentration in dialysis fluid ([Mg]d) plus postdilution and predilution flows times fluid´s magnesium concentration ([Mg]d). The same fluid was used for dialysis, postdilution and predilution.

Magnesiumflux=(Qd×[Mg]d+postdilution×[Mg]d+predilution×[Mg]d)−Qeff×[Mg]eff

The calcium flux was calculated similarly, i.e. deducting the calcium amount eliminated in the effluent from the calcium input. The effluent amount of calcium was calculated as Qeff multiplied by the effluent calcium concentration ([Ca]eff). Due to the fact that dialysate and postdilution/predilution calcium concentrations ([Ca]d) equal zero the calcium input was calculated as calcium replacement flow (Q[Ca]) times 10% calcium chloride concentration ([Ca]in = 0.456 mmol/ml). For better comparison to magnesium flux the results were recorded as calcium flux without postfilter calcium substitution and overall calcium balance including postfilter CaCl_2_ infusion.

Calciumflux=Q[Ca]×[Ca]in−Qeff×[Ca]eff

The fluxes and balances of both ions were tested for correlations with dosages of citrate, dosage of citrate per blood flow and with changes of circuit citratemias (i.e. absolute value of difference between prefilter and postfilter levels of citrate).

The statistical analysis was performed using Statistica v.9 software. Data sets were checked for distribution and the differences between groups were evaluated using Mann-Whitney U test. Correlations between various parameters were tested using Pearson´s test. Comparisons between multiple sets of measured parameters were performed with Kruskal-Wallis ANOVA. Statistical significances were set at 0.05 and 0.01 levels.

## Results

45 critically ill patients (22 CVVHDF and 23 CVVH) were started on fluid containing magnesium 0.75 mmol/l and entered the study after at least one day on CRRT. All patients were mechanically ventilated and had a renal indication to CRRT. Their mean age was 64.1±12.8, admission APACHE II was 28.0±7.6. The first sampling (T0(Mg0.75)) was after a median of 49.5 (36–94) h in CVVHDF and 36 (24–65) h in the CVVH patients. Three patients (profound septic shock in two and pulmonary embolism with surgery in one) did not complete the 27-hour study (terminated after 15, 19 and 25 hours) due to metabolic acidosis with extreme hyperlactataemia or prolonged surgery. Altogether 42 patients (21 CVVHDF and 21 CVVH) completed the study after the switch to fluid containing Mg of 1.50 mmol/l. Clotting occured in 17 blood circuits of the 45 included patients, i.e. occured during 27h on the top of median of 49.5h of running circuits before study started in CVVHDF patients and of 36h in CVVH patients. The median filter life with current setting was 64 (43–108)h. When clotting occured the filters and circuits were immediately replaced and the study continued.

**Fig 3:**
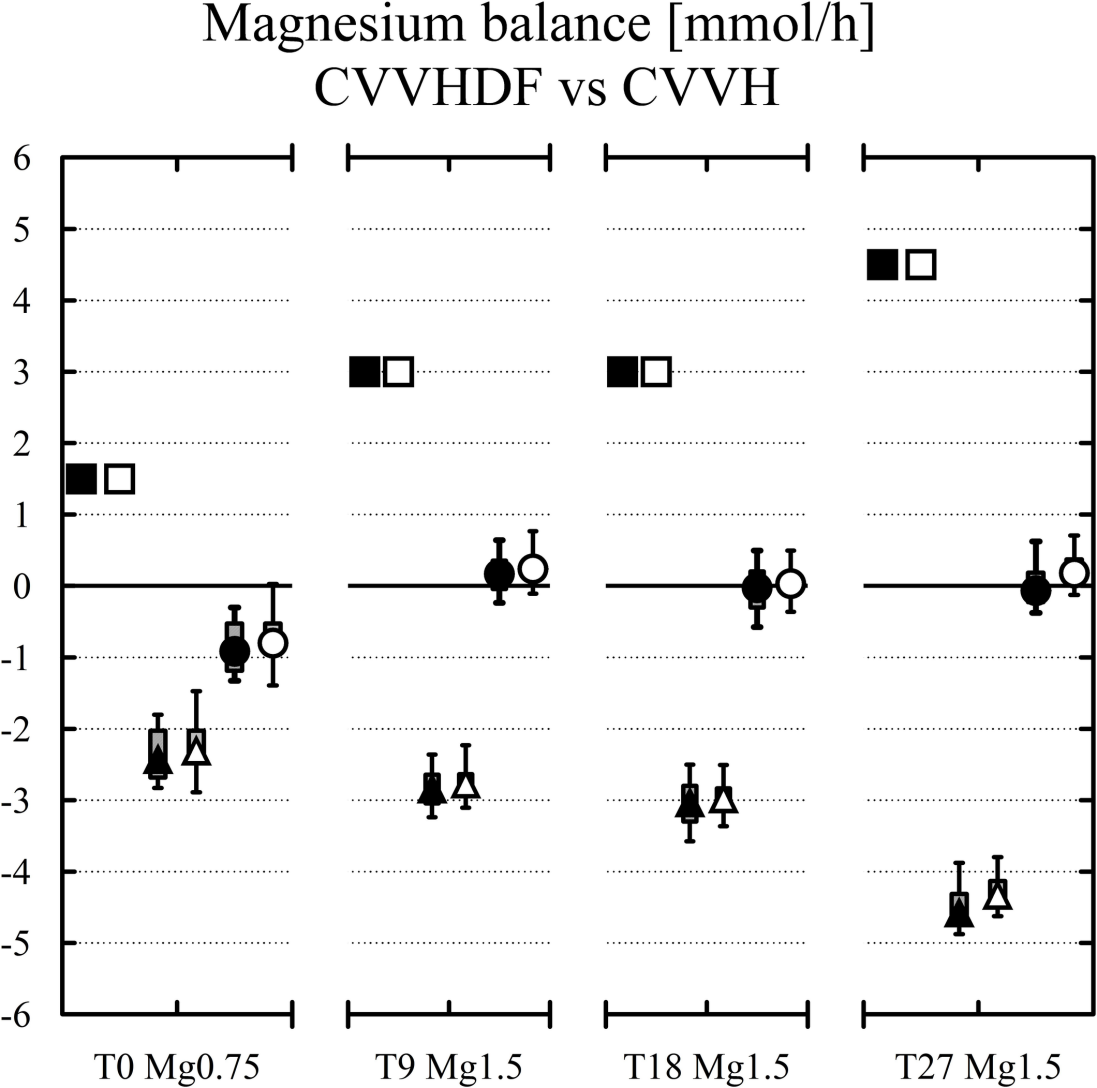
Magnesium inputs (black squares for CVVHDF and white squares for CVVH, both above zero) vs magnesium losses (black triangles for CVVHDF and white triangles for CVVH). The final balances at each study time (black and white circles) are in the middle. Median, IQR in boxes, Min-Max in whiskers.

**Fig 4:**
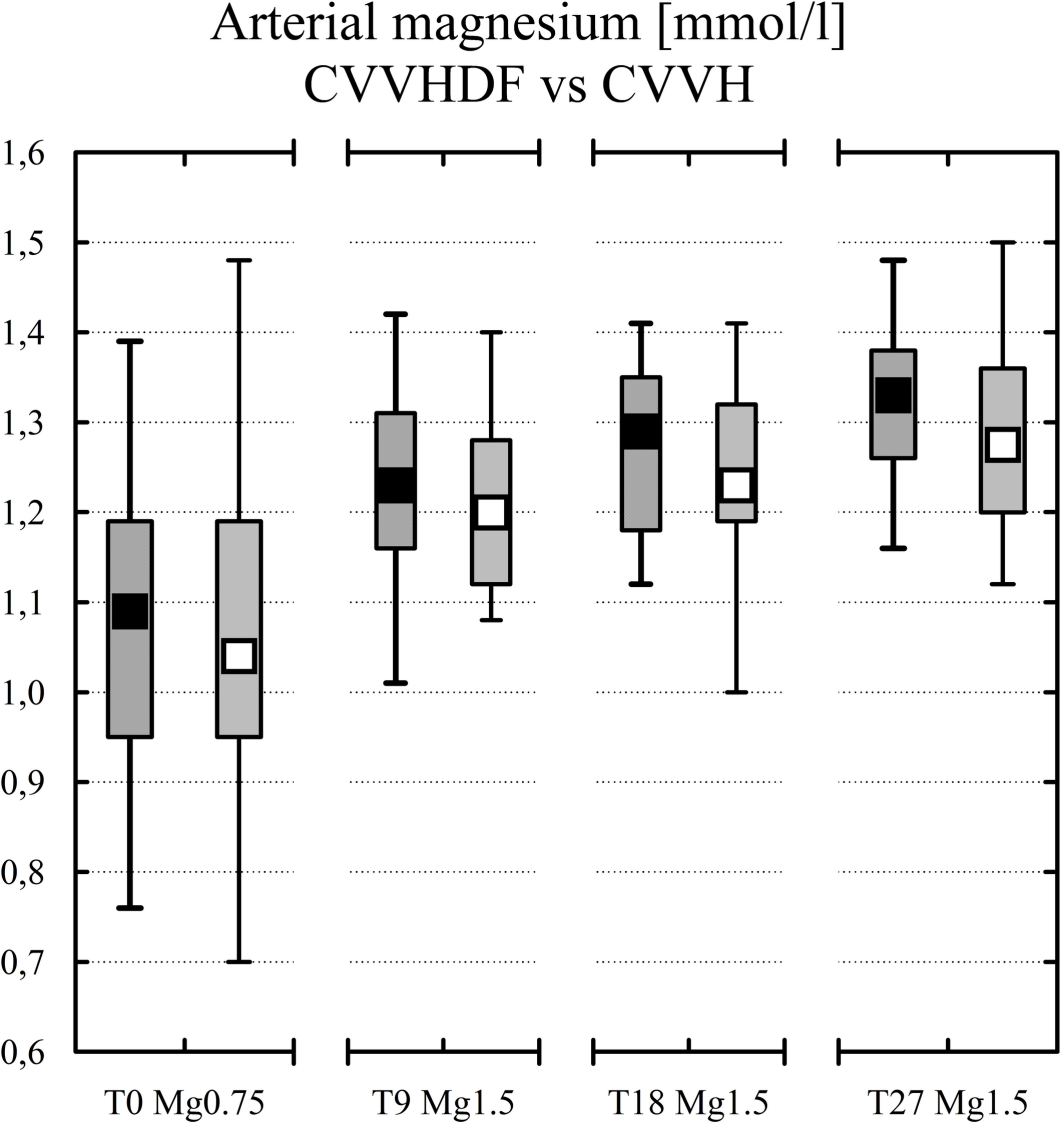
Arterial Mg levels in CVVHDF (black squares), CVVH (white squares) at each study time. Median, IQR in boxes, Min-Max in whiskers.

**Fig 5:**
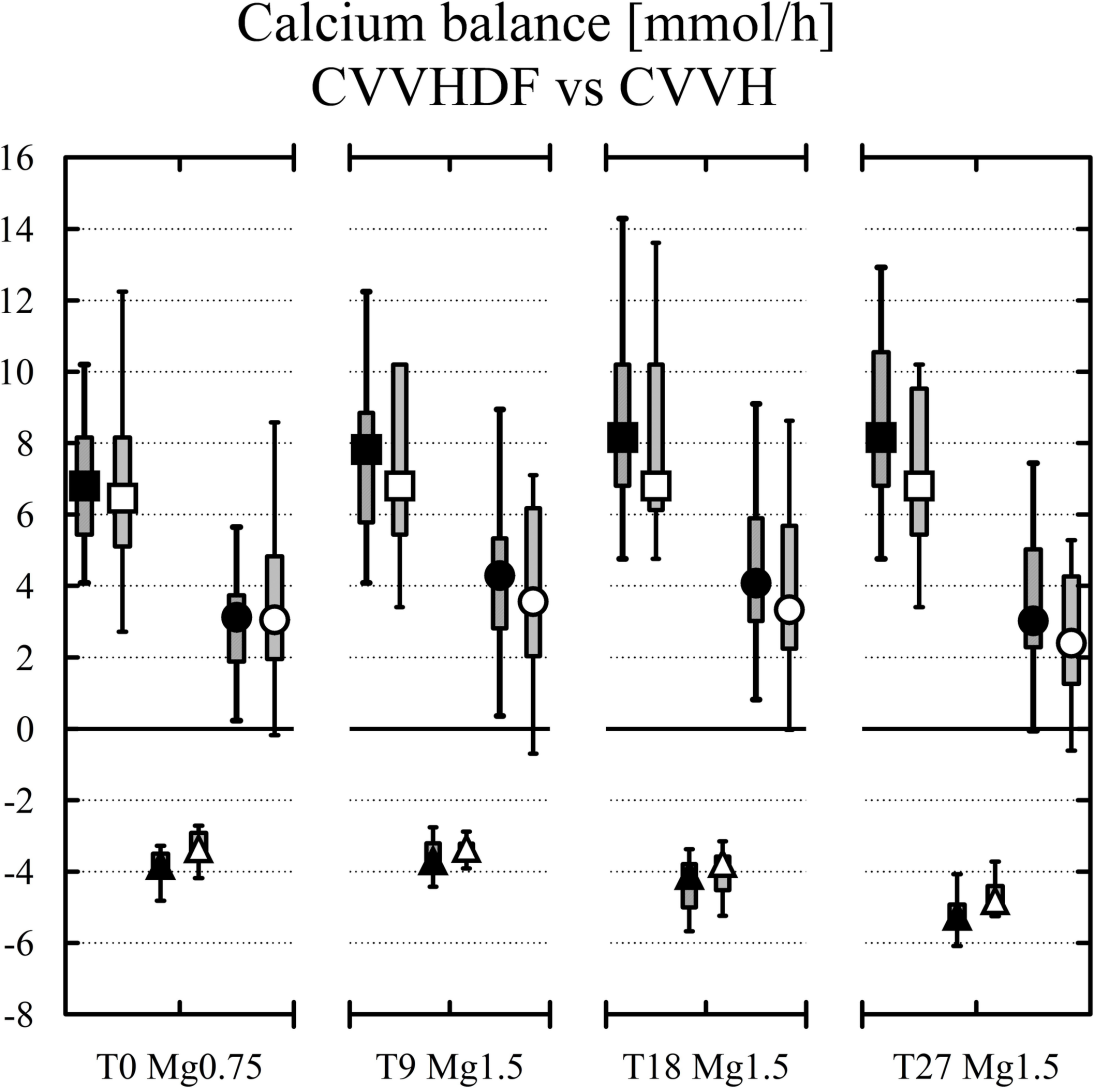
Calcium inputs (black squares for CVVHDF and white squares for CVVH, both above zero) vs calcium losses (black triangles for CVVHDF and white triangles for CVVH). The final balances at each study time (black and white circles) are in the middle. Median, IQR in boxes, Min-Max in whiskers.

The results for magnesium and calcium are given separately for CVVH and CVVHDF in Table 1. The magnesium balance, levels of arterial magnesium and calcium balance are shown in Figs 3, 4 and Fig 5. There was a general absence of differences between the two most frequent CRRT modalities (see Table 1) except for the post to pre-filter citrate concentration difference in the CVVH subgroup (see Table 1) and for the Mg balance at T27 (see Table 1). An explanation for the higher citrate difference at two samplings in CVVH subgroup lies in lower prefilter citrate concentration due to predilution in CVVH and to countercurrent of dialysate solution (no citrate) and blood lowering postfilter citratemia in CVVHDF [27].

**Table 1:** The results (median, IQR) are given separately for the CVVHDF (the first row) and CVVH (the second row) subgroups. Differences between CVVHDF and CVVH reached statistical significance for post to pre-filter citrate difference (p<0.05) at T9(Mg1.50), T27(Mg1.50) and for Mg balance at T27(Mg1.50) (p = 0.02). Comparisons between T0(Mg0.75) and T9(Mg1.50), T18(Mg1.50), T27(Mg1.50) are given separately for CVVHDF and CVVH where statistically significant.

CVVHDF (n = 22) CVVH (n = 23)	T0(Mg0.75): Qb 100 ml/min 2000 ml/h	T9(Mg1.50): Qb 100 ml/min 2000 ml/h	T18(Mg1.50): Qb 150 ml/min 2000 ml/h	T27(Mg1.50): Qb 150 ml/min 3000 ml/h
4%TSC dose [ml/h]	200 (180–230)	200 (180–233)	300 (248–350)^**A**^^,^^**B**^	300 (240–345)^**A**^^,^^**B**^
	200 (180–220)	200 (190–230)	305 (243–338)^**A**^^,^^**B**^	260 (250–345)^**A**^^,^^**B**^
Dose of 4%TSC/Qb [mmol/l.h]	4.4 (4.1–5.4)	4.5 (4.1–5.3)	4.5 (3.7–5.3)	4.5 (3.6–5.2)
	4.5 (4.1–5.2)	4.5 (4.3–5.2)	4.6 (3.7–5.1)	3.9 (3.8–5.2)
Post to pre- filter citrate [mmol/l]	3.49 (2.57–4.5)	2.90 (2.62–3.47)	3.29 (2.32–3.91)	3.51 (2.45–3.86)
	4.25 (3.46–5.11)	4.25 (3.64–4.86) (p<0.05)	3.75 (3.32–4.52)	3.92 (3.30–4.72) (p<0.05)
Citrate filter removal [%]	49.9 (43.8–54)	47.1 (40.4–53)	34.4 (29–36.3)^**A**^^,^^**B**^	47.5 (38.9–54)^**C**^
	46.8 (39.9–49.6)	41.9 (37.5–49.5)	33.9 (31–37.2)^**A**^^,^^**B**^	43.1 (38.8–47)^**C**^
Ca_tot_ [mmol/l]	2.27 (2.07–2.47)	2.05 (1.78–2.4)	2.31 (1.83–2.75)	2.07 (1.82–2.37)
	2.1 (1.82–2.46)	2.01 (1.86–2.31)	2.16 (1.87–2.52)	1.96 (1.79–2.16)
Ca^2+^ [mmol/l]	1 (0.87–1.08)	1.04 (0.97–1.1)	1.02 (0.93–1.11)	0.98 (0.93–1.04)
	1 (0.97–1.05)	1.05 (1–1.08)	1.06 (0.95–1.12)	0.96 (0.92–1.02)
Ca index [Ca_tot_/Ca^2+^]	2.12 (1.95–2.35)	1.97 (1.83–2.18)	2.26 (1.97–2.48)	2.11 (1.95–2.28)
	2.12 (1.88–2.34)	1.98 (1.87–2.14)	2.04 (1.97–2.26)	2.04 (1.95–2.12)
Ca balance [mmol/h]	3.12 (1.89–3.74)	4.29 (2.89–5.17)	4.09 (3.16–5.71)	3.02(2.46–4.86)
	3.05 (1.97–4.78)	3.57 (2.24–6.16)	3.34 (2.24–5.69)	2.40 (1.26–4.27)
Mg_tot_ [mmol/l]	1.09 (0.95–1.19)	1.23(1.16–1.31)^**A**^	1.29(1.18–1.35)^**A**^	1.33(1.26–1.38)^A,D^
	1.04 (0.95–1.19)	1.2 (1.13–1.28)^**E**^	1.23 (1.2–1.31)^**A**^	1.28 (1.2–1.36)^**A**^^,^^**D**^
Mg balance [mmol/h]	-1.02 (-1.2 to -0.54)	0.17(-0.02–0.32)^**A**^	-0.02 (-0.23–0.19)^**B**^	-0.06 (-0.20–0.18)^**B**^
	-0.80 (-0.91 to -0.56)	0.24(0.09–0.35)^**A**^	-0.03(-0.05 to -0.17)^**B**^	0.18 (0.03–0.37)^**E**^

^A^ significantly different from T0, p<0.01

^B^ significantly different from T9, p<0.01

^C^ significantly different from T18, p<0.05

^D^ significantly different from T9, p<0.05

^E^ significantly different from T0, p<0.05

The overall lack of differences between CVVHDF and CVVH allowed also to present the results for all 42 patients. The magnesium balance was negative -0.81 (-1.16 to -0.53) mmol/h when using fluid with Mg 0.75 mmol/l (T0(Mg0.75)) compared to 0.2 (0.06–0.35) mmol/h under fluid with Mg 1.50 mmol/l and the same setting of CRRT (T9(Mg1.50), p<0.001). When increasing blood flow and dosage of citrate to a median of 300 ml/h (243–350), p<0.001 [21] the balance of magnesium was close to zero (0.02 (-0.12–0.18), T18(Mg1.50), p<0.001). Then with the increased dosage of high magnesium containing fluid (3000 ml/h) at T27(Mg1.50) the hourly balance of magnesium was mildly positive (0.15 (-0.11–0.25) mmol/h, p<0.001).The development of Mg level in arterial blood is shown in Fig 4. The box plots show magnesium at the upper limit of normal (1.05 (0.95–1.19) mmol/l) when ordinary Mg 0.75 mmol/l containing fluid was applied. Mild hypermagnesemia developed (1.21 (1.14–1.30) mmol/l) when the same setting was tested with the fluid containing Mg 1.50 mmol/l (p<0.01). The level was not different to Mg level (1.27 (1.19–1.33) mmol/l) in higher Qb and dosage of citrate and to the level (1.31 (1.22–1.37) mmol/l) in higher dosage of the novel fluid.

The balances of calcium (Fig 5) were significantly more positive compared to magnesium (Fig 3, all p<0.01) which can be explained by calcium parenteral substitution as part of the RCA protocol. The initial calcium balance under fluid with Mg 0.75 mmol/l was positive 3.12 (1.94–4.15) mmol/h similarly to 3.88 (2.73–5.9) mmol/h in the same setting with fluid containing Mg 1.50 mmol/l (ns). The balance was not different (3.68 (2.47–5.74) mmol/h, ns) using fluid with Mg 1.50 mmol/l with increased blood and citrate flows. The calcium balance was less positive (2.76 (1.53–4.57) mmol/h, ns) with Mg 1.50 mmol/l fluid and the increased dosage of CRRT, i.e. with increased flow of calcium free fluid.

The dosages of citrate per blood flow and circuit differences of citratemias did not change throughout the study (Tab.1). Neither changes of Mg nor Ca were related to the dosages of citrate, dosage of citrate per blood flow and to changes of circuit citratemias.

## Discussion

The present prospective observational cohort study shows that the RCA associated loss of magnesium is not covered by ordinary levels of magnesium in ordinary calcium free fluid and is higher when higher blood flow and dosage of citrate are used. CRRT with RCA may contribute to factors leading to hypomagnesemia in critically ill patients [1, 8, 9].

The novel fluid with higher magnesium levels showed negligibly positive magnesium balance under RCA only when limited blood flow and citrate dosage was applied (estimated +4.8 mmol of Mg^tot^/day) or when CRRT dosage of 3000 ml/h was used (+3.6 mmol of Mg^tot^/day). An increase of Qb to 150 ml/h with high dosage of 4%TSC (median 300 ml/h) reduced positive balance of magnesium to +0.48 mmol of Mg^tot^/day due to higher loss of magnesium-citrate complexes in the effluent.

The presence of magnesium in dialysis/substitution fluid in citrate anticoagulated CRRT is a matter of debate because Mg is also chelated by citrate which may neutralize a portion of citrate and increase the demand for its infusion to lower Ca^2+^ postfilter into desired range. The importance of removing magnesium for reduction of filter clotting is questionable and eventual lowering of citrate dosage does not outweight the risk of hypomagnesemia if Mg is not replenished properly [8, 18, 19]. Magnesium is the second most important intracellular ion with many important physiological functions. The data suggest that improperly magnesium substituted citrate CRRT may contribute to depletion of its rather low body pool of 1000 mmols. The extrapolated daily loss of magnesium is still more than recommended daily allowances for magnesium [6]. The utilization of citrate for anticoagulation of RRT has been on the rise since the introduction of new automated citrate-calcium modules, at the same time about 10% of general population present with a magnesium deficit which is far more frequent in ICU patients and reaches almost 60% [8]. Magnesium deficit has multiple pathophysiologic consequences however, the efficacy of routine magnesium substitution in critically ill misses strong evidence [1].

Our study suffers from several limitations. First, the study was not randomized. Second, the cohorts were sampled only once. The calculated daily loss of Mg is therefore an estimate, because continuous is not always continuous [28]. We did not correct for filter-down time, which differs between centers and modalities. Estimates of total loss of Mg may therefore be 10–20% lower. However, this applies less for the RCA, because circuit life with citrate was longer [29, 30]. Third, with regard to studies dealing with the homeostatic effects of various fluids, we assume that 27 h divided into 9-hour steps is enough to support our conclusions [21]. High flux CVVH with lactate-based fluid demonstrated stable biochemical parameters after 6 h with a steady state during the last 2 h of the 8-hour cross-over interval [31]. The metabolic impact of a lactate-based fluid was tested with 24-hour cross-over design [32] and an observational protocol similar to this study demonstrated stabilisation of the biochemical parameters after 12–16 h with little change beyond 24 h [16]. Fourth, the limitation of using different buffers in fluids of this study can be seen as an opportunity to show that the elimination of magnesium during citrate CRRT in general depends on the blood flow used, the amount of citrate applied, and the level of magnesium in the dialysis/replacement fluids. All patients of both study and control groups had renal indication to CRRT and were treated with the same devices, tubings and filters, the same postfilter calcium endpoint of prefilter citrate dosage, the same regimen of calcium substitution and initially with the same dosage of CRRT. The proportion of citrate to anticoagulated blood was the same in both RCA groups. The exact concentrations of citrate per l liter of blood flow within the blood circuit were not significantly different (Tab.1). Fifth, the level of magnesium in the novel fluid might rise a question in terms of risk of mildly positive magnesium balance. Our feasibility testing suggests that this would concern long term CRRT application and would be likely offset by effects of RCA [7, 9]. Moreover, certain patients may benefit from mild hypermagnesemia like cardiovascular, cardiosurgical and neurosurgical. Preeclampsia and eclampsia require reaching high level magnesium which is rather unlikely using just dialysis/substitution fluid with the level of 1.50 mmol/l. Meticulous approach require neuromuscular disease like myasthenia gravis where hypermagnesemia is not desirable. Sixth, the statistically significant yet clinically irrelevant difference of Mg balance between CVVHDF and CVVH at the time of higher dosage of the novel fluid should be clarified by further research focusing an optimal fluid magnesium dose.

## Conclusions

In conclusion, the present study shows that the loss of Mg in RCA with conventional fluids is not balanced by the concentrations in commercially available dialysis/substitution fluids. Citrate anticoagulation of CRRT may lead to depletion of the bodies magnesium pool with potential for organ dysfunction. The difficult assessment of intracellular magnesium particularly in intensive care calls for magnesium loading tests [6] to reveal a possible magnesium deficit under continuous citrate anticoagulation. Standard low level of magnesium in substitution fluid does not prevent development of hypomagnesemia during citrate CRRT which increases daily requirements for magnesium to extra 2 to 3 grams of MgSO_4._ This requirement may increase with CRRT dosage above 20–25 ml/kg.h. A risk of magnesium depletion seems to be avoided with the novel fluid [21] which has magnesium level doubled (1.50 mmol/l) compared to the ordinary fluids used in intensive care however, validation and safety require further research. The fluid provided magnesium levels with no extra replenishment other than magnesium content in ordinary commercially available enteral and parenteral nutrition.
